# Iodine(V)-Based Oxidants in Oxidation Reactions

**DOI:** 10.3390/molecules28135250

**Published:** 2023-07-06

**Authors:** Samata E. Shetgaonkar, Subhiksha Jothish, Toshifumi Dohi, Fateh V. Singh

**Affiliations:** 1 School of Chemical Sciences, Goa University, Taleigao Plateau 403206, Goa, India; samatashetgaonkar@gmail.com; 2 Chemistry Division, School of Advanced Sciences (SAS), Vellore Institute of Technology, Chennai 600127, Tamil Nadu, India; 3 College of Pharmaceutical Sciences, Ritsumeikan University, 1-1-1 Nojihigashi, Kusatsu 525-0058, Shiga, Japan

**Keywords:** hypervalent iodine(V) reagents, oxidant, catalyst, oxidation

## Abstract

The chemistry of hypervalent iodine reagents has now become quite valuable due to the reactivity of these compounds under mild reaction conditions and their resemblance in chemical properties to transition metals. The environmentally friendly nature of these reagents makes them suitable for Green Chemistry. Reagents with a dual nature, such as iodine(III) reagents, are capable electrophiles, while iodine(V) reagents are known for their strong oxidant behavior. Various iodine(V) reagents including IBX and DMP have been used as oxidants in organic synthesis either in stoichiometric or in catalytic amounts. In this review article, we describe various oxidation reactions induced by iodine(V) reagents reported in the past decade.

## 1. Introduction

Hypervalent iodine reagents are environment friendly tools for the construction of simple and complex organic molecules [1,2,3,4,5,6,7,8]. These reagents are potential oxidants due to their excellent oxidizing and electrophilic properties [9,10,11,12,13]. The unique characteristics attributed to these reagents are non-toxicity, easy handling, high reactivity and stability, combined with good site selectivity and broad applicability in several synthetic transformations [14,15,16]. Therefore, hypervalent iodine reagents are of paramount importance to organic chemistry for the development of new asymmetric and non-asymmetric reactions. In particular, hypervalent iodine compounds are the reagents of choice for oxidation reactions [17,18,19,20], cyclizations [21,22,23], rearrangements [24,25,26], *a*-functionalization of carbonyl compounds [27,28], atom-transfer reactions [29] and alkene difunctionalizations reactions [30,31]. Recently, the application of these reagents has been successfully expanded to organocatalysis [32,33,34,35,36,37], C−H bond functionalization [38], stereoselective synthesis [39,40] and photochemical reactions [41,42].

**Figure 1:**
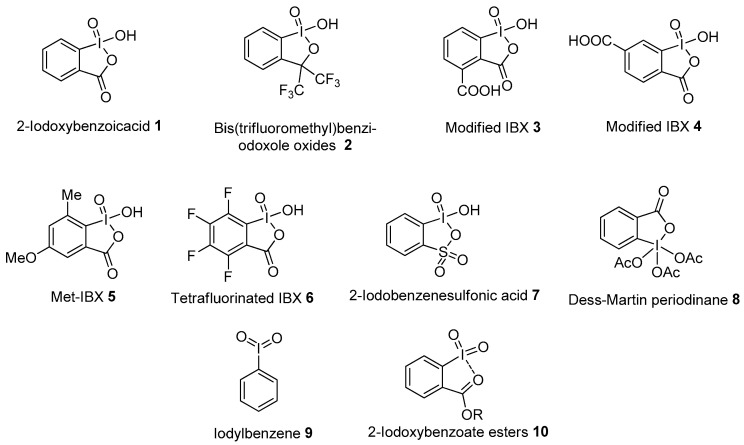
Hypervalent iodine(V) reagents **1**−**10**.

In recent years, the chemistry of hypervalent iodine(V) compounds has witnessed considerable growth in comparison to that of trivalent iodine reagents, as reviewed by Zhdankin in 2006 [43] and 2011 [44]. Some examples of common hypervalent iodine(V) reagents are presented in Figure 1. The most versatile hypervalent iodine(V) reagent is *o*-iodoxybenzoic acid (IBX **1**), first synthesized by Hartmann and Meyer in 1893 [45]. Later, Mullins’s research group synthesized IBX **1** through the oxidation of 2-iodobenzoic acid using potassium bromate under acidic conditions [46]. However, the presence of bromate impurities imparted an explosive nature to IBX **1** under excessive heating conditions. In addition, the practical use of IBX **1** as a potential oxidant was overlooked for many years due to its poor solubility in most organic solvents except DMSO. Nevertheless, IBX has received renewed attention after the pioneering work by Santagostino et al. regarding its improved synthesis t from 2-iodobenzoic acid in the presence of oxone in an aqueous medium [47]. Since then, IBX **1** has become the main representative of hypervalent iodine chemistry owing to its unique reactivity and excellent oxidizing properties. The numerous IBX-mediated chemical transformations include the oxidation of alcohols to carbonyl compounds, the oxidation of amines, the oxidation of benzylic carbon and the oxidation of phenols [43,44]. IBX-mediated oxidative cyclization reactions giving access to diverse heterocycles have also been well explored over the years [48].

In order to solve solubility issues, several analogs of IBX were prepared by functionalizing its aromatic core. Dess and Martin synthesized the stable, non-explosive bis(trifluoromethyl)benziodoxole oxide **2** having good solubility in many organic solvents [49]. Later, the water-soluble modified IBX (mIBX) **3** and **4** were prepared from terephthalic acid by Thottumkara and Vinod for the oxidation of benzylic and allylic alcohols [50,51]. Furthermore, Moorthy and co-workers designed and synthesized the *ortho*-methyl-substituted IBX (Me-IBX, **5**) that oxidizes alcohols in common organic solvents [52]. Then, Wirth and co-workers introduced a novel tetrafluorinated IBX analogue (FIBX **6**), which has higher solubility and reactivity than IBX **1** [53]. Zhdankin’s group prepared 2-iodobenzenesulfonic acid (IBS **7**) from 2-iodobenzenesulfonic acid using Oxone in aqueous solution [54]. This thia-IBX **7** was eventually used by Ishihara and co-workers for the oxidation of alcohols [55]. Another interesting iodine(V) reagent is Dess–Martin periodinane (DMP **8**), mainly used for the oxidation of primary alcohols to aldehydes and secondary alcohols to ketones [56,57]. Among acyclic iodine(V) reagents, iodylbenzene **9** is the most explored and is well suited for the oxidation of phenols, sulfides and alcohols [43,44]. Recently, Motlagh and Zakavi synthesized, characterized and studied the oxidizing strength of iodylbenzene nanofibers for the oxidation of 1,5-dihydroxynaphthalene to juglone [58]. Besides this, pseudocyclic iodine(V) compounds **10** are also important oxidants having the characteristic of establishing intramolecular secondary I---O bonding interactions between the iodine center and the oxygen atom in the *ortho* substituent [59,60]. The present review article summarizes the recent advances in oxidative transformation reactions such as the oxidation of alcohols, amines, amides, aromatic compounds and oxidative cyclizations using hypervalent iodine(V) reagents. Moreover, recent developments achieved in the design of catalytic systems based on in situ generated hypervalent iodine(V) reagents from corresponding iodoarenes will be discussed in great detail. 

## 2. Oxidation of Alcohols

**Scheme 1:**
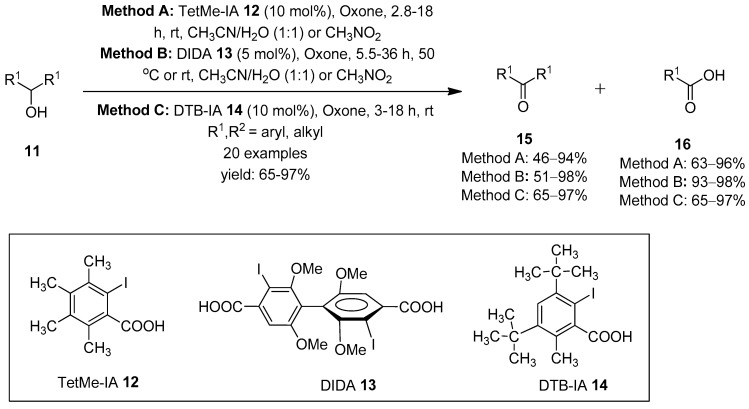
Oxidation of alcohols **11** to carbonyl compounds **15** and **16** using iodo-acids **12**–**14** as precatalysts in the presence of Oxone.

Carbonyl compounds (aldehydes, ketones, carboxylic acids, esters, amides, lactones, etc., are versatile building blocks in organic chemistry [61,62]. The oxidation of alcohols to the corresponding carbonyl compounds have been well explored using hypervalent iodine(V) reagents as stoichiometric oxidants [43,44]. The explosive nature and low solubility of IBX in organic solvents stimulated researchers to develop catalytic routes involving the in situ generation of hypervalent iodine(V) species from organoiodo compounds in the presence of a suitable co-oxidant. Within this context, in 2005, Thottumkara et al. successfully achieved the catalytic oxidations of alcohols by generating iodine(V) species in situ from *o*-iodobenzoic acid in the presence of Oxone as an oxidant in the solution state [63]. Later in 2009, Ishihara et al. employed *o*-iodobenzenesulfonate (IBS) **7** as a catalyst to produce iodine(V) species for the oxidation of alcohols to the corresponding carbonyl compounds in good yields [55]. Furthermore, Moorthy’s research group accomplished significant achievements in the catalytic oxidation of alcohols using different iodoarenes as precatalysts. Initially, they employed 3,4,5,6-tetramethyl-2-iodobenzoic acid (TetMe-IA) **12** as an iodo-acid precursor for the in situ generation of reactive TetMe-IBX that facilitated the oxidation of alcohols **11** to carbonyl compounds **15** at room temperature (Scheme 1) [64]. Notably, primary alcohols **11** were oxidized to carboxylic acids **16** through the oxidation of initially formed aldehydes by Oxone. Further catalytic oxidation of a variety of diols to the corresponding lactones was achieved using TetMe-IA **12** as a precursor of TetMe-IBX [64]. 

Later, the same group generated the Bis-IBX catalyst in situ from twisted 3,3′-diiodo-2,2′,6,6′-tetramethoxybiphenyl-4,4′-dicarboxylic acid (DIDA) **13** for the catalytic oxidation of alcohols **11** [65]. Furthermore, Mishra and Moorthy recently designed and synthesized a catalyst, 3,5-di-*tert*-butyl-2-iodobenzoic acid (DTB-IA) **14**, for the in situ generation of IBX **1** using Oxone as a terminal oxidant (Scheme 1) [66]. The highly reactive DTB-IA **14** was then used to oxidize a variety of primary, secondary, aliphatic and aromatic alcohols **11** in solid state under ball-milling conditions. 

**Scheme 2:**
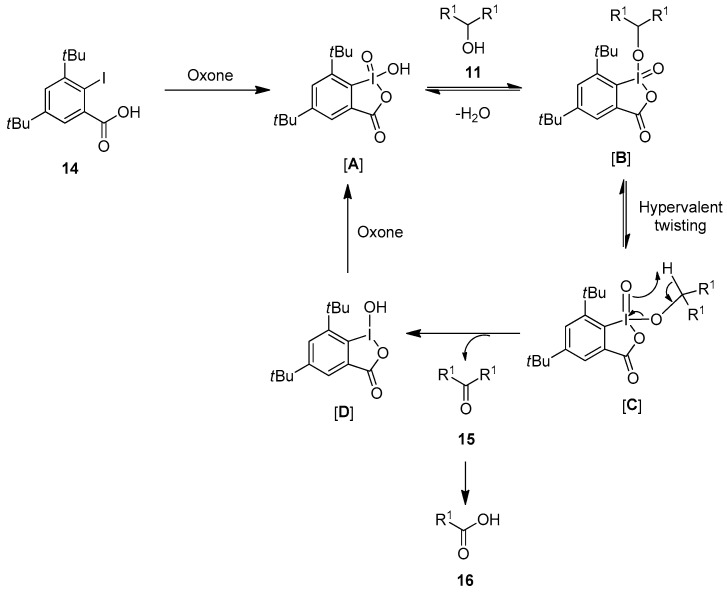
Catalytic cycle for the oxidation of alcohols **11** to carbonyl compounds **15** using the iodo-acids **14** as precatalysts in the presence of Oxone.

The reaction mechanism for the oxidation of alcohols **11** to carbonyl compounds **15** using the precatalysts **12**–**14** in the presence of Oxone is depicted in Scheme 2. The reaction proceeds with the in situ oxidation of iodarene **14** to the iodine(V) intermediate [**A**], which reacts with the alcoholic substrate **11** to form another intermediate [**B**]. Furthermore, the intermediate [**B**] undergoes a process called hypervalent iodine twisting and produces the intermediate [**C**]. Finally, the intermediate [**C**] undergoes a reductive elimination and yields the final product along with the formation of the iodine(III) intermediate [**D**]. Furthermore, the iodine(III) intermediate [**D**] oxidizes to an active iodine(V) species [**A**] in the presence of Oxone to continue the catalytic cycle. Notably, final product **15** could be further converted into to carboxylic acids **16** through the oxidation of initially formed aldehydes by Oxone.

**Scheme 3:**
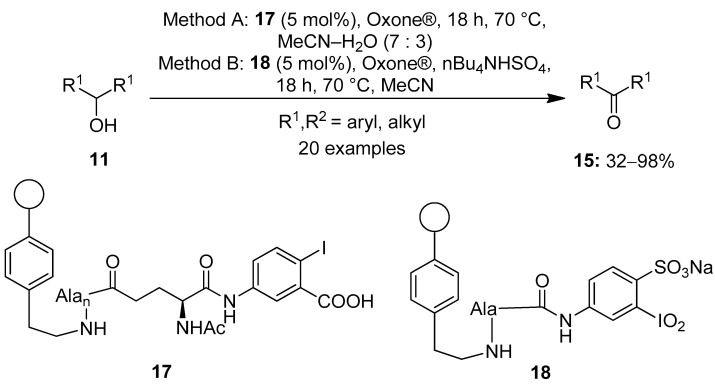
Oxidation of alcohols **11** to carbonyl compounds **15** using solid-supported iodoarenes **17** and **18** in the presence of a terminal oxidant.

Meanwhile, Ballaschk and Kirsch performed the oxidation of secondary alcohols **11** to produce ketones **15** using the inexpensive and recyclable solid-supported hypervalent iodine catalysts **17** and **18** in the presence of stoichiometric amounts of Oxone^®^ (Scheme 3) [67]. In this, the hypervalent iodine precursor was connected by stable amide bonds to the aminoethyl polystyrene resin. The catalysts were easily regenerated by simple filtration, and the activity lasted for five rounds. Both IBX-derived (Method A) and IBS-derived (Method B) catalytic systems yielded a variety of structurally diverse carbonyl compounds **15** in good to excellent yields. *^n^*Bu_4_NHSO_4_ was used as phase transfer catalyst in non-aqueous conditions (method B). Notably, the IBS-derived catalyst **18** was found to be more active and provided higher product yields compared to **17**. Secondary alcohols which were sterically hindered provided better yields when method B was used.

**Scheme 4:**
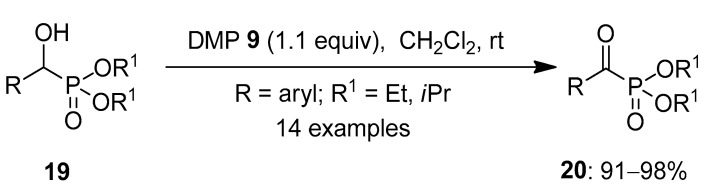
Oxidation of α-hydroxyphosphonates **19** to *α*-ketophosphonates **20** using DMP **8** as an oxidant.

In 2021, Kupwade et al. worked towards synthesizing α-ketophosphonates **20** by oxidizing α-hydroxyphosphonates **19** in the absence of metal catalysts (Scheme 4) [68]. Usually, *o*-iodoxybezoic acid **1** (IBX) is used for the oxidation of alcohols. However, it was found to be inefficient for the oxidation of such compounds. Later, IBX **1** in combination with benzyltriphenylphosphonium peroxymonosulfate (BTPP) in the ratio of 1:3 was used, and this resulted in excellent yields. The major limitation for this technique was the high molecular weight, cost and reflux conditions of BTPP. Hence, Dess–Martin periodinane (DMP) **8** and **19** in the ratio of 1:1 were stirred together for a very short time, resulting in the desired products **20** under ambient conditions. The reagent when tested with several α-hydroxyphosphonates **19** substituted with electron-donating and -withdrawing groups furnished **20** in 91–98% yield. Notably, α-ketophosphonates exhibits interesting biological activities [69,70] and are versatile molecules in organic synthesis [71]. 

## 3. Oxidation of Amines 

**Scheme 5:**
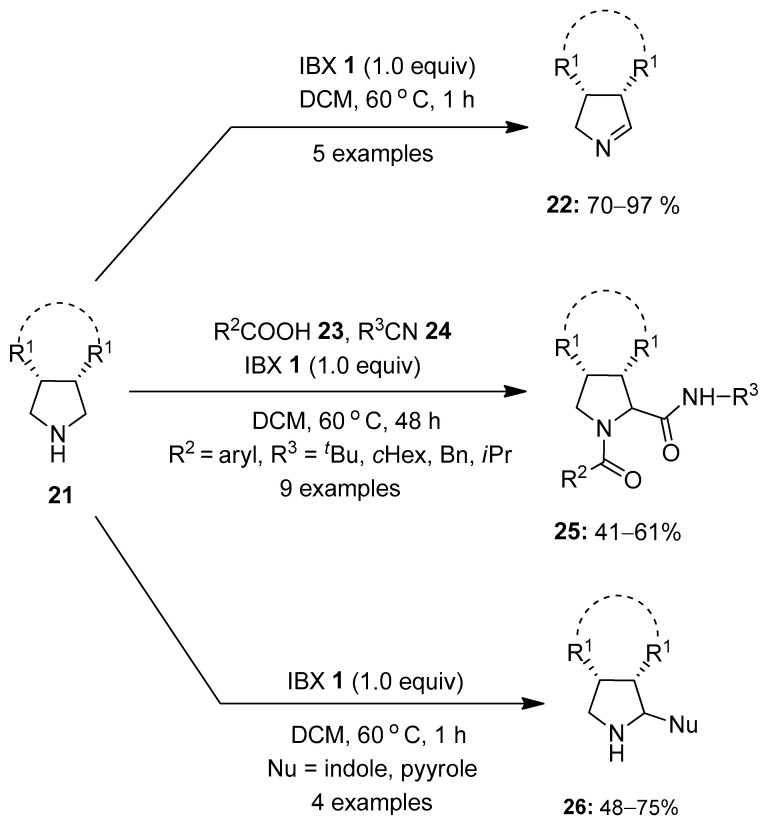
Oxidation of unactivated amines **21** to imines **22** and 2-substituted pyrrolidines **25**/**26** using IBX **1** as an oxidant.

The oxidation of amines using hypervalent iodine reagents has attracted great attention in recent years [72,73,74]. Recent accomplishments achieved in this area using iodine (V) reagents are reported in this section. In 2015, Orru and co-workers for the first time reported the oxidation of unactivated amines **21** to the corresponding imines **22** using IBX **1** as an oxidant (Scheme 5) [75]. Delightedly, a number of aliphatic *meso*-pyrrolidines **21** were oxidized selectively by IBX **1** to furnish bi- and tricyclic 1-pyrrolines **22** in 70–97% yield. Furthermore, a one-pot Ugi-type three-component reaction between *meso*-pyrrolidines **21**, carboxylic acids **23** and isocyanides **24** produced dipeptides **25** as a single diastereoisomer in moderate to good yields (41–61%). The molecular diversity of in situ-generated 1-pyrrolines **22** was further explored through the oxidative aza-Friedel–Crafts reaction of *meso*-pyrrolidines **21** with pyrrole and indoles, providing 2-substituted pyrrolidines **26** in useful yields.

**Scheme 6:**
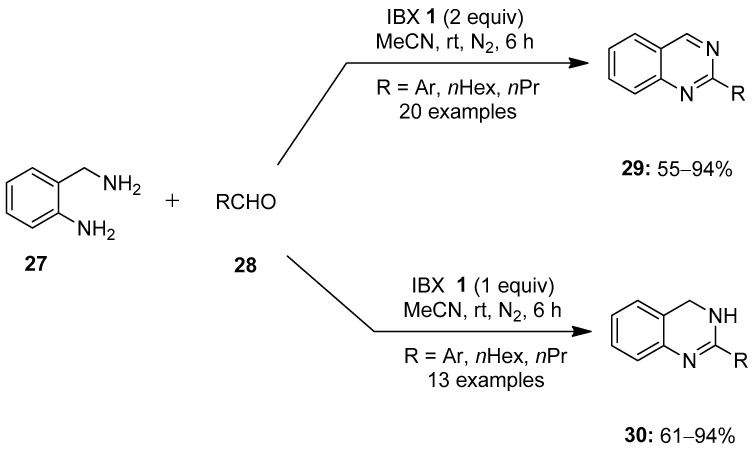
Synthesis of quinazolines **29** and 3,4-dihydroquinazolines **30** from *o*-aminobenzylamine **27** with aldehydes **28** using IBX **1** as an oxidant.

In 2016, Hati and Sen reported a facile method for the synthesis of functionalized quinazolines **29** and 3,4-dihydroquinazolines **30** via an IBX-mediated tandem reaction of *o*-aminobenzylamine **27** with aldehydes **28** (Scheme 6) [76]. Notably, the reaction with two equivalents of IBX **1** yielded quinazolines **29,** while one equivalent of IBX **1** provided dihydroquinazolines **30**. This strategy was found effective for a number of aryl, heteroaryl and alkyl aldehydes and also tolerated both electron-donating and -withdrawing functional groups.

**Scheme 7:**
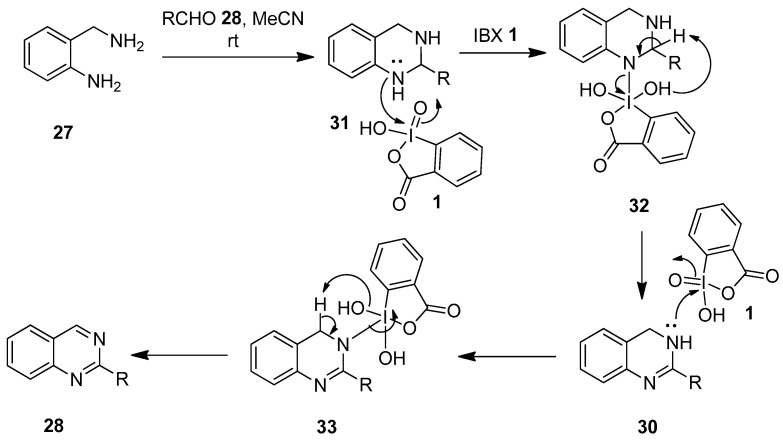
Plausible mechanism for the synthesis of quinazolines **28** and 3,4-dihydroquinazolines **30** using IBX **1** as an oxidant.

A plausible mechanism envisioned for the synthesis of quinazolines **29** and 3,4-dihydroquinazolines **30** is depicted in Scheme 7 [76]. Initially, *o*-aminobenzylamine **27** reacts with aldehydes **28** to form the tetrahydroquinazoline intermediate **31**, which attacks the electrophilic iodine center of IBX **1** to produce intermediate **32**. Subsequently, the reduction of **32** generates the dihydroquinazoline **30**. Finally, the IBX-mediated oxidation of **30** through intermediate **33** yields the desired quinazoline **28**. 

**Scheme 8:**
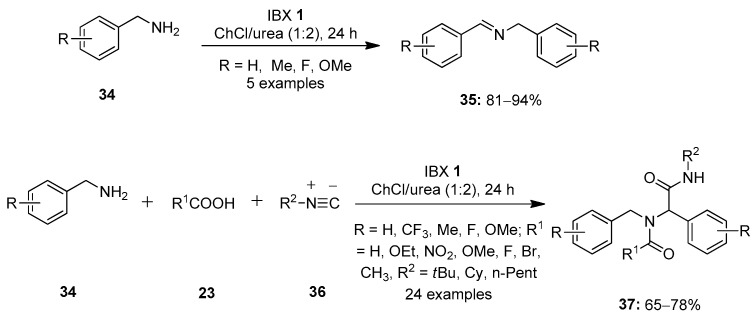
Oxidation of primary amines **34** to imines **35** using IBX **1** as an oxidant.

In 2019, Singh et al. reported a methodology to selectively oxidize the primary amines **34** to the corresponding imines **35** using IBX **1** as an oxidant (Scheme 8) [77]. It was found that for oxidative coupling, IBX **1** and DMP **8** were highly selective. Due to the high solubility of IBX **1** in the deep eutectic solvent choline chloride/urea (ChCl/urea), this solvent is used as solvent system for this reaction. A number of electron-rich and electron-deficient amines were readily converted into secondary imines in good yields. Notably, IBX **1** and the solvent could be recovered and reused up to five times without loss of much activity. Further, a one-pot three-component Ugi reaction involving the condensation of diverse carboxylic acids **23** and primary amines **34** was carried out to form the imine intermediate **35** followed by a reaction with an isocyanide **36** to yield bis(amide)s **37** in high yields.

**Scheme 9:**
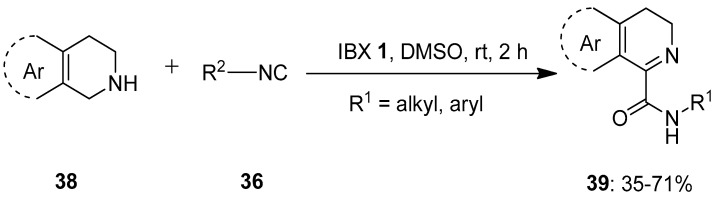
Oxidative addition of isocyanides **36** to the cyclic amines **38** to yield imino-carboxamides **39** using IBX **1** as an oxidant.

In 2019, Ambule et al. reported a pioneering work on the IBX-mediated oxidative addition of isocyanides **36** to the cyclic amines **38** such as tryptolines and 1,2,3,4-tetrahydroisoquinolines to obtain imino-carboxamides **39** under metal-free conditions (Scheme 9) [78]. The dual role of IBX **1** as an oxidant and as a Lewis acid to activate an imine facilitates the isocyanide addition in this transformation. A variety of aliphatic and aromatic isocyanides **36** reacted well with **38** to afford products **39** in good to moderate yields. However, the reactions with unactivated secondary amines such as pyrrolidine, piperidine, epoxyisoindoline and indoline were sluggish due to the formation of a complex mixture of products. Furthermore, this method was successfully employed for the gram-scale preparation of two alkaloids, alangiobussine (63%) and alangiobussinine (45%).

**Scheme 10:**
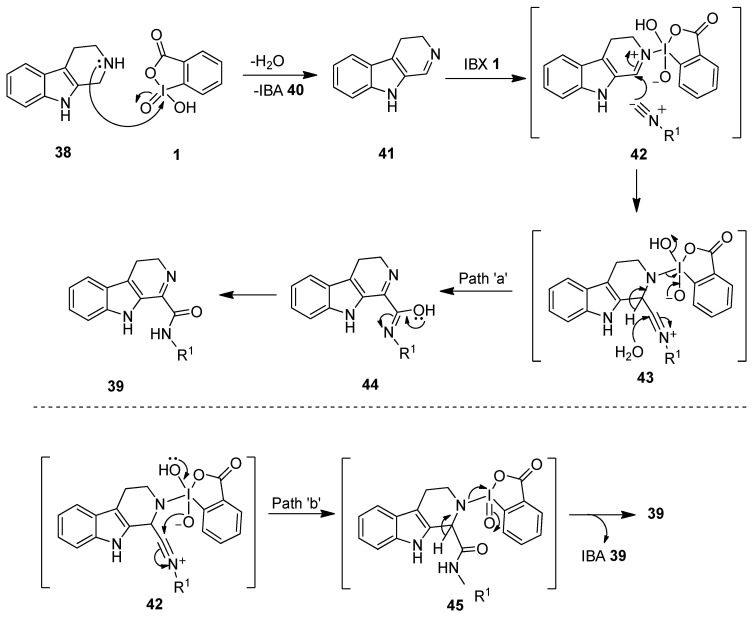
The proposed mechanism for the synthesis of imino-carboxamides **39** using IBX **1** as an oxidant.

A proposed mechanistic pathway for this reaction is displayed in Scheme 10 [78]. Initially, IBX oxidizes tryptoline **38** to 3,4-dihydro-*β*-carboline **41**, followed by the imine activation with another molecule of IBX **1** or IBA **40** to yield the intermediate **42**. Notably, the activation of the imine by IBX **1** facilitates the isocyanide **36** addition to the intermediate **42** to form the nitrilium ion **43**. Product **39** could be obtained from the intermediate **43** via two routes (paths a and b). In path ‘a’, the addition of water to the intermediate **43** yields product **39** via the intermediate **44** (Path a). In path ‘b’, the intermediate **43** undergoes an intramolecular hydroxyl transfer to form **45**, which provides product **39** upon loss of IBA **40**. 

## 4. Oxidative Cleavage of Amides

**Scheme 11:**
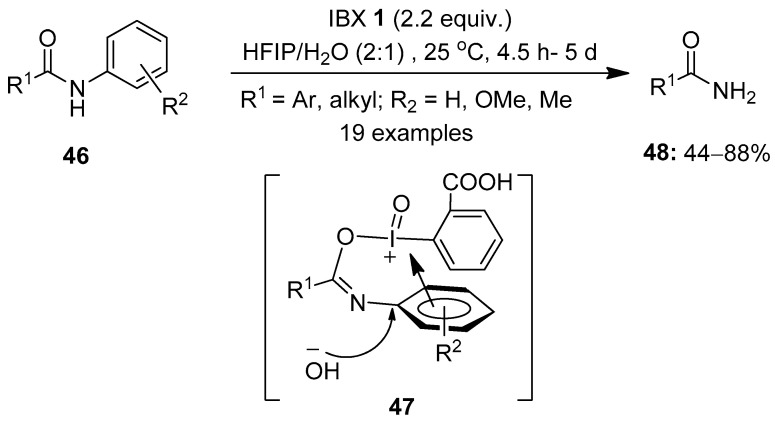
Oxidative cleavage of aryl C−N bonds in *N*-aryl amides **46** to yield primary amides **48** using IBX **1** as an oxidant.

Another interesting area of great interest is the oxidation of amides using hypervalent iodine(V) reagents. In 2018, Zhang et al. demonstrated an excellent method for the oxidative cleavage of inert aryl C−N bonds in *N*-aryl amides **46** to yield primary amides **48** using IBX **1** as an oxidant (Scheme 11) [79]. Among the different solvent systems screened, HFIP/H_2_O was found to be very efficient for these reactions. The plausible mechanism involves the interaction of IBX with substrates **46** to form the annular π-complex **47**, which is subsequently attacked by the hydroxy group obtained from H_2_O to provide the primary amide **48** via a regioselective cleavage of the C(aryl)−N bond. Notably, substrates with electron-donating groups yielded products in good yields, whereas those with electron-withdrawing groups failed to provide the desired products. The key aspect of this method is that IBX enables the selective cleavage of the C(aryl)−N bond in *N*-aryl amides, keeping the C(carbonyl)−N bond untouched. Furthermore, this novel strategy was extended to a number of *α*-mono- and *α*,*α*-disubstituted *β*-ketoamides **46** to yield the anticipated amides **48** in useful yields.

**Scheme 12:**
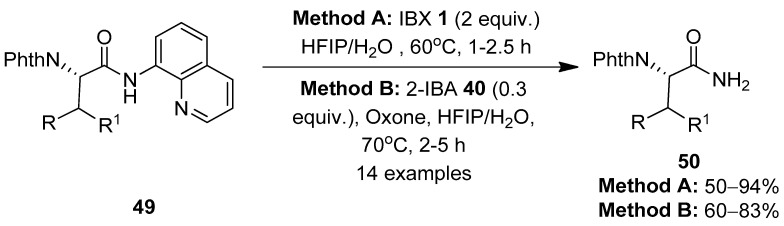
Oxidative cleavage of 8-aminoquinoline in *N*-quinolyl carboxamides **49** to yield primary amides **50** using IBX **1** as an oxidant.

The same group reported a chemoselective method for the oxidative cleavage of 8-aminoquinoline (AQ) in *N*-quinolyl carboxamides **49** and the removal of the AQ group using IBX **1** as a stoichiometric oxidant (Method A) (Scheme 12) [80]. The reaction scope was evaluated with a variety of AQ-coupled substrates, and the corresponding primary amide products **50** were obtained in moderate to good yields. The mixture of HFIP and H_2_O solvents in a 1:1 ratio was critical for obtaining high yields of products. An additional catalytic system (Method B) comprising 2-iodobenzoic acid **40** (0.3 equiv.) and Oxone (a mixture of 2KHSO_5_·KHSO_4_·K_2_SO_4_) as a co-oxidant successfully furnished products **50** in comparable amounts to those obtained with method A. Notably, the reactions exhibited excellent chemoselectivity towards the *C*-terminal *N*-quinolyl carboxamide, without affecting the internal alkyl amide groups. Finally, the resulting primary amides **50** were easily converted into carboxylic acids by treating with *tert*-butyl nitrite in AcOH.

## 5. Oxidation of Alkenes

**Scheme 13:**
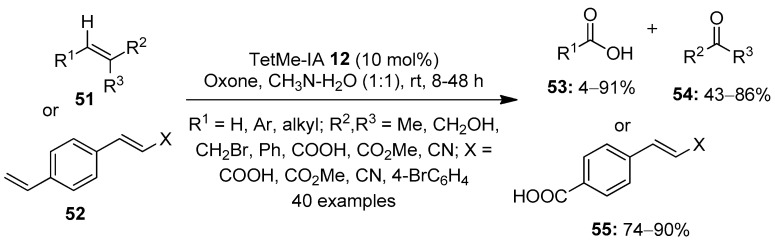
Oxidative cleavage of alkenes **51** or **52** into ketones **54**/carboxylic acids **53** using TetMe-IA **12** as a precatalyst in the presence of Oxone.

The selective oxidation of alkenes to more polar compounds using hypervalent iodine reagents is yet another interesting area of research. In 2014, Moorthy’s research group demonstrated the oxidative cleavage of alkenes **51** or **52** into ketones **54**/carboxylic acids **53** using a catalytic amount of TetMe-IA **12** in the presence of Oxone (Scheme 13) [81]. Mechanistically, the reaction proceeds via the initial dihydroxylation of alkenes followed by oxidative cleavage by the in situ generated TetMe-IBX to aldehydes, which undergo a rapid oxidation with Oxone to produce the corresponding acids **53**. The reaction was carried out with a variety of terminal and internal alkenes, and the desired products were obtained in respectable yields. Notably, for substrates containing two double bonds, chemoselective cleavage of electron-rich alkenes was observed.

**Scheme 14:**
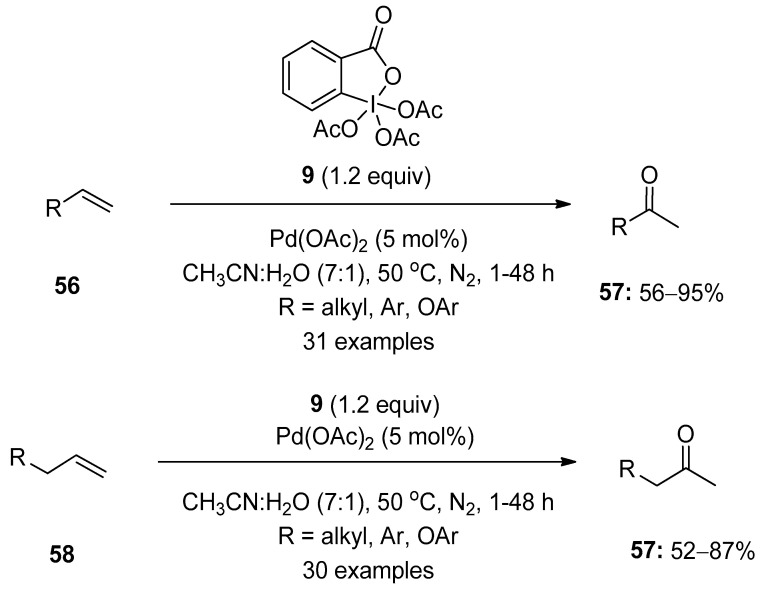
Pd(II)-catalyzed Wacker-type oxidation of alkenes **56** and **58** to ketones **57** using DMP **8** as an oxidant.

Chaudhari and Fernandes reported the palladium-catalyzed Wacker-type oxidation of terminal alkenes **56** using Dess–Martin periodinane (DMP) **8** as an oxidant (Scheme 14) [82]. This operationally simple method enabled the synthesis of diverse methyl ketones **57** in good yields with complete Markonikov selectivity. Additionally, allylic or homoallylic compounds **58** were oxidized to methyl ketones **57** under similar conditions. The key features of this reaction are its broad substrates scope, excellent functional group tolerance and high yields.

## 6. Oxidation of Aromatic Compounds

**Scheme 15:**
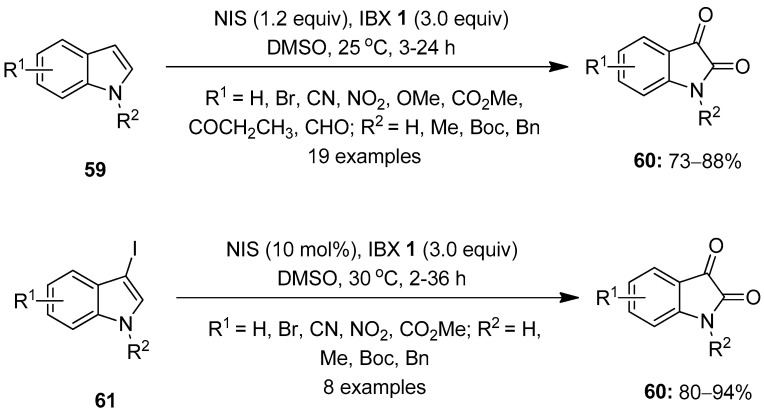
Oxidation of indoles **59** and 3-iodoindoles **61** to isatins **60** using IBX **1** as an oxidant.

The hypervalent iodine-mediated oxidation of aromatic compounds has been well studied by several researchers. In 2006, Moorthy and co-workers reported a one-pot oxidation of stilbene derivatives to the corresponding benzils with NIS/IBX in DMSO [83]. In continuation, Moorthy’s research group demonstrated a method for the direct oxidation of indoles **59** to isatins **60** using the NIS/IBX **1** reagent in DMSO at room temperature (Scheme 15) [84]. The reactions of a variety of substituted indoles **59** proceeded smoothly under the optimized conditions, providing isatins **60** in good yields. Notably, the reaction proceeds through the formation of the intermediary 3-iodoindole **61**, which is oxidized by IBX **1** to produce isatins **60**. Furthermore, 3-iodoindoles **61** were synthesized independently by reacting indoles **59** with NIS and efficiently converted into isatins **60** with IBX **1**. A similar method was developed for the synthesis of isatins by Kirsch and others using a NaI/IBX-SO_3_K reagent mixture [85].

**Scheme 16:**
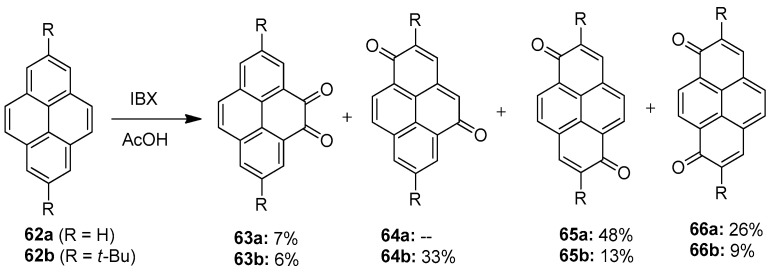
Oxidation of pyrenes **62** to diones **64**–**66** using IBX **1** as an oxidant.

The oxidation of the K-region (4,5,9,10 position) in pyrene **62** is found to be very difficult, as most of the oxidants produce very less yields (Scheme 16). El-Assaad et al. reported a new method for this reaction, using an hypervalent iodine compound, in 2020 [86]. They performed the oxidation of pyrene **62** by employing IBX **1** as an oxidizing agent in acetic acid. A mixture of diones **63**–**66** was obtained, and *pseudo*-para-diones were found to be the major product (diones **65a** and **64b**).

**Scheme 17:**
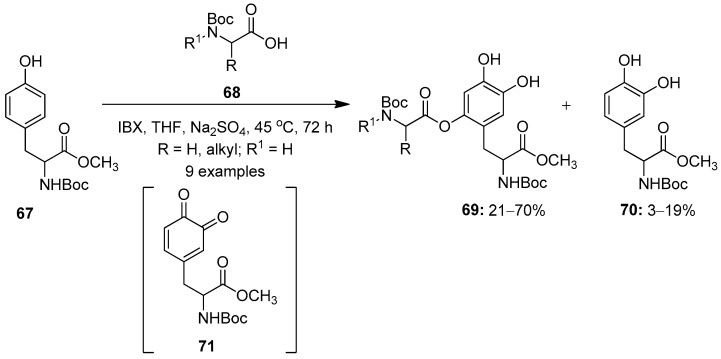
Oxidative functionalization of tyrosine **67** to L-DOPA-peptidomimetics **69** using IBX **1** as an oxidant.

Meanwhile, Saladino’s research group synthesized DOPA peptidomimetics **69** by the aromatic oxidative functionalization of the tyrosine molecule **67** with IBX **1** (Scheme 17) [87]. The reactions proceeds through the oxidation of tyrosine by IBX **1** to form the DOPA quinone intermediate **71**, followed by a Michael-like nucleophilic addition of nitrogen-protected amino acids **68** to yield new L-DOPA-peptidomimetics **69** along with the formation of the side product **70**. A further oxidative functionalization of tyrosine **67** with O-protected α-amino acids was achieved under similar conditions.

**Scheme 18:**
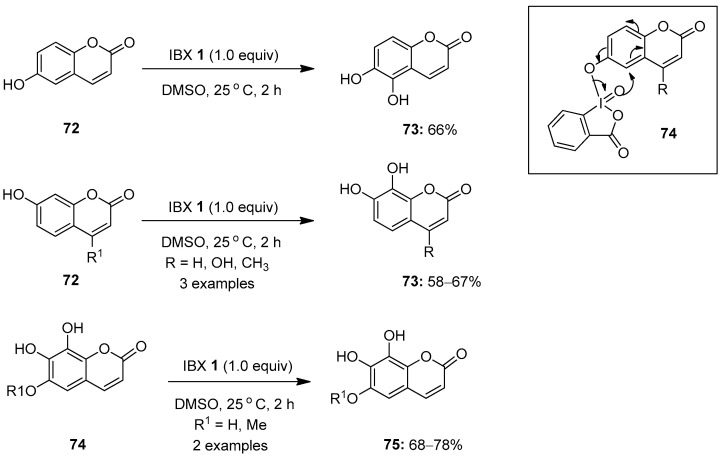
Oxidation of coumarins **72** and **74** to catechols **73** and pyrogallol derivatives **75** using IBX **1** as an oxidant.

In continuation, Nencioni, Saladino and co-workers eventually reported the IBX-mediated oxidation of coumarins **72** in DMSO for the regioselective synthesis of catechols **73** (Scheme 18) [88]. Additionally, the synthesis of pyrogallol derivatives **75** was achieved through the oxidation of fraxetin and esculetin **74** under similar conditions, in good yields. Notably, the regioselectivity observed in this transformation is due to the intramolecular delivery of the oxygen atom from the λ^5^-iodanyl intermediate **74** to the ortho-position of the phenolic moiety. Moreover, the oxidation of coumarins was also achieved by replacing IBX **1** with polystyrene-supported IBX in the presence of water as a solvent. Finally, the synthesized coumarin derivatives were tested for antioxidant and antiviral activities, and the corresponding pyrogallols **75** were found to be the most active compounds. 

## 7. C−H Functionalization Reactions

**Scheme 19:**
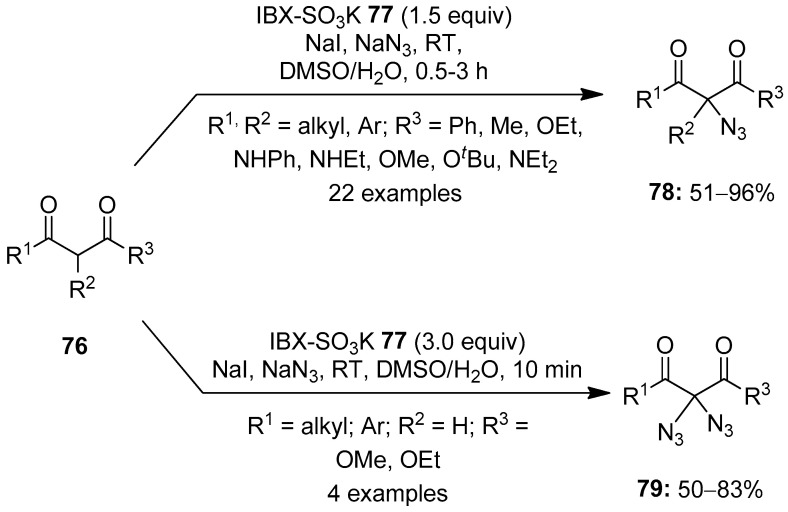
Azidation of 1,3-dicarbonyl compounds **76** to the azide products **78** and **79** using iodine(V) reagent **77** in combination with NaI.

The C−H functionalization of organic compounds has emerged as a powerful tool to access biologically and pharmaceutically important molecules. The use of hypervalent iodine(V) reagents in C−H functionalization reactions is well studied, and the recent advancements in this area will be discussed in this section. In 2012, Klahn and others reported an operationally simple method for the azidation of 1,3-dicarbonyl compounds **76** using NaN_3_ as an azide source (Scheme 19) [89]. The reaction proceeds in the presence of 2-iodoxybenzoic acid (IBX)-SO_3_K **77**/NaI as an oxidant. The present azidation protocol exhibited a broad substrates scope and tolerated a multitude of functional groups. Furthermore, 1,3-dicarbonyl compounds **76** with no substituent at the 2 position (R^2^ = H) smoothly underwent a novel double azidation reaction to furnish 2,2-bisazido-1,3-dicarbonyl compounds **79** in good yields under slightly modified conditions. Moreover, the azidation of two natural products, *β*-estradiol and strychnine, was achieved under these conditions in useful yields.

**Scheme 20:**
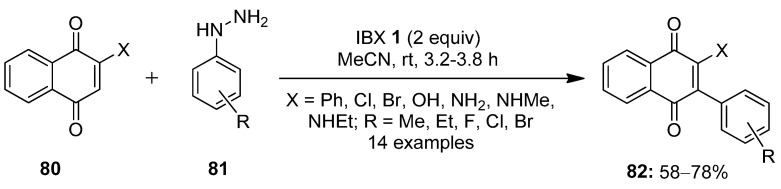
C−H arylation of naphthoquinones **80** using arylhydrazines **81** as an aryl source and IBX **1** as an oxidant.

In 2014, Akamanchi and co-workers reported an excellent method for the arylation of naphthoquinones **80** with arylhydrazines **81** using IBX **1** as an oxidizing agent (Scheme 20) [90]. The combination of arylhydrazines **81** and IBX **1** facilitates the in situ generation of aryl free radicals, which act as the aryl source. The reactions went smoothly with a number of substituted naphthoquinones **80** and arylhydrazine derivatives **81**. Electronically diverse arylated naphthoquinones **82** were isolated in moderate to good yields under mild conditions. The synthetic utility of arylated naphthoquinones **82** was demonstrated through the short and high-yielding synthesis of benzocarbazoledione, an important antitumor–antibiotic precursor. Previously, the same group reported the *N*-arylation of aromatic amines using a combination of arylhydrazines and IBX [91].

**Scheme 21:**
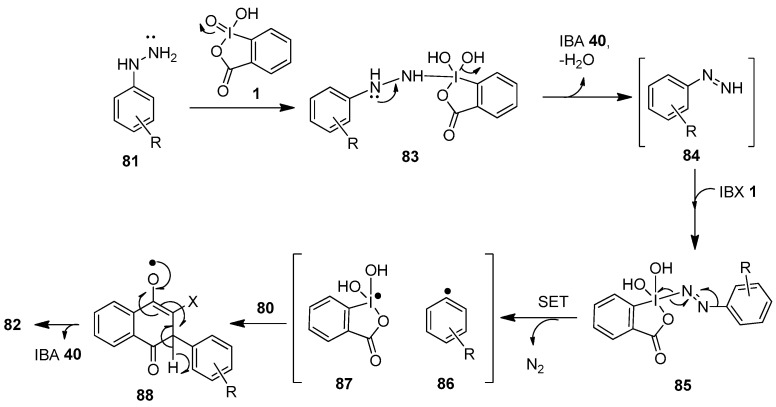
A proposed mechanism for the IBX-mediated C−H arylation of naphthoquinones **80** using arylhydrazines **81** as an aryl source.

A postulated radical-mediated mechanism for the C−H arylation of naphthoquinones **80** is displayed in Scheme 21 [90]. In the beginning, IBX **1** oxidizes arylhydrazine **81** to generate the intermediate **83**, which loses a water molecule to produce phenyldiazine **84** and IBA **40**. Then, the nucleophilic phenyldiazine **84** attacks another IBX **1** molecule to form the intermediate **85**, which later undergoes oxidative cleavage to yield the phenyl radical **86** and the species **87** through single-electron transfer (SET). Finally, the phenyl radical **86** attracts the electrophilic C-2 or C-3 positions of naphthoquinone **80** to form the intermediate **88**, which provides the arylated product **82** with the release of IBA **40** and water.

**Scheme 22:**
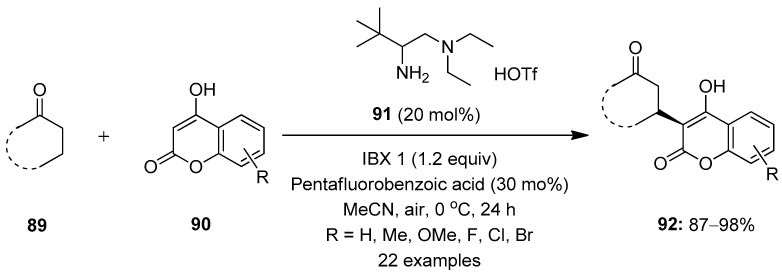
Enantioselective β-C−H functionalization of simple ketones **89** using IBX **1** as an oxidant.

Zhu et al. reported a method involving the enantioselective *β*-C−H functionalization of simple ketones **89** with coumarins in the presence of the chiral primary amine **91** as a catalyst under mild oxidizing conditions, with IBX **1** as an oxidant (Scheme 22) [92]. The reaction was carried out at 0 °C in the presence of acetonitrile as a solvent. The weak acid-like additive pentafluorobenzoic acid was necessary for high reactivity and enantioselectivity. Using these conditions, cyclic and acyclic ketones **89** smoothly underwent reactions, furnishing chiral ketones **92** with *β*-stereocenters. A number of electron-rich and -deficient coumarins **90** as nucleophiles tolerated the reaction and produced good yields. However, the *β*-C−H functionalization of cyclopentanone failed, and no product formation was observed under these conditions.

## 8. Oxidative Cyclization Reactions

**Scheme 23:**
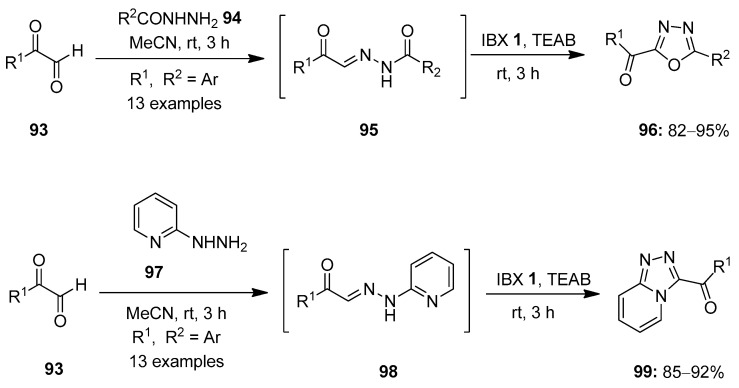
Oxidative cyclization of hydrazide-hydrazones **95** to α-keto-1,3,4-oxadiazoles **96** and α-keto-1,2,4-triazolo [4,3-a]pyridines **99** using IBX **1** as an oxidant.

Hypervalent iodine(V) reagents have been widely used in oxidative cyclization reactions owing to their excellent electrophilic character. Several heterocycles including benzimidazoles, benzoxazoles, 1,3,4-oxadiazoles, imidazoles, imidazo-pyridines, thiazoles, thiazolines, etc., were synthesized using IBX **1** as an oxidant [93,94,95,96,97]. In this section of the review, we will discuss recent work conducted on the oxidative cyclization reactions mediated by iodine(V) reagents. In 2014, Kumar and co-workers prepared α-keto-1,3,4-oxadiazoles **96** via IBX-mediated oxidative cyclization of hydrazide-hydrazones **95** generated in situ from arylgyloxals **93** and hydrazides **94** (Scheme 23) [98]. The use of tetraethylammonium bromide (TEAB) as an additive was necessary to activate IBX **1**. The key features associated with this method were high yield, mild conditions, gram -cale synthesis, short reaction times and broad functional group tolerance. Furthermore, α-keto-1,2,4-triazolo [4,3-a]pyridines **99** were synthesized from arylglyoxals **93** and 2-hydrazinopyridines **97** under the same reaction conditions.

**Scheme 24:**
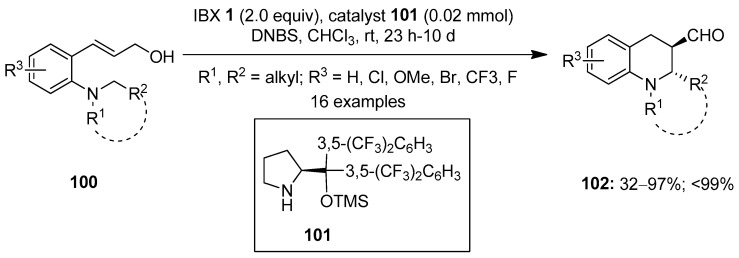
Oxidative coupling of 3-arylprop-2-en-1-ols **100** to yield chiral tetrahydroquinolines **102** using IBX **1** as an oxidant.

In 2015, Kim and co-workers described the synthesis of chiral tetrahydroquinolines **102** via IBX-mediated enantioselective intramolecular oxidative coupling of 3-arylprop-2-en-1-ols **100** using 2,4-dinitrobenzensulfonic acid (DNBS) **101** as a catalyst (Scheme 24) [99]. Both electron-withdrawing and electron-donating substituents were tolerated in **100**, and the corresponding products **102** were isolated with excellent enantioselectivity and up to 99% *ee*. The reaction was proposed to proceed via the oxidation of **100** using IBX **1** followed by 1,5-hydride transfer/ring closure to yield the desired products **102**.

**Scheme 25:**
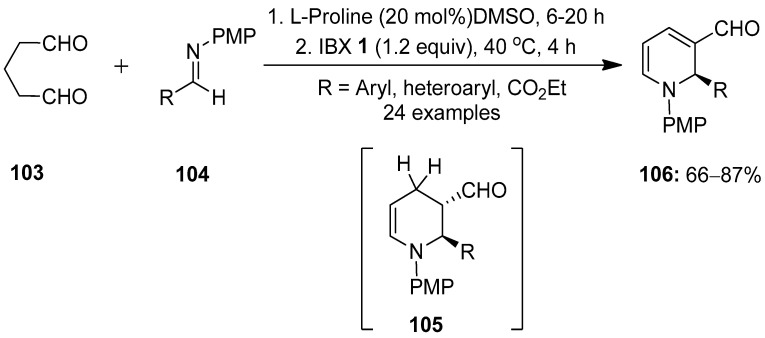
IBX-mediated synthesis of N-PMP-1,2-dihydropyridines (DHPs) **106** via the proline-catalyzed [4 + 2] cycloaddition of glutaraldehyde **103** with aldimines **104**.

In continuation, Kumar and co-workers reported the enantioselective proline-catalyzed synthesis of N-PMP-1,2-dihydropyridines (DHPs) **106** via a one-pot [4 + 2] cycloaddition reaction (Scheme 25) [100]. This chemistry, involving the L-proline-catalyzed direct Mannich reaction/cyclization between glutaraldehyde **103** and aldimines **104** generating tetrahydropyridines **105** in situ, followed by IBX-mediated oxidation, led to the synthesis of DHPs **106**. The practical utility of this method was demonstrated through the gram-scale synthesis of N-PMP-1,2-DHPs **106** and the rapid synthesis of a fused chiral tetrahydroquinoline-based skeleton.

**Scheme 26:**
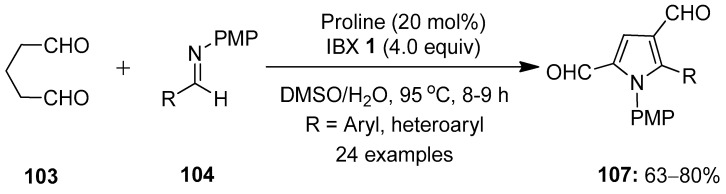
IBX-mediated synthesis of pyrrole-2,4-dialdehydes **107** by reacting glutaraldehyde **103** with N-(4-methoxyphenyl)aldimines **104** using proline as a catalyst.

The same group developed an interesting approach for the preparation of pyrrole-2,4-dialdehydes by treating glutaraldehyde **103** with N-(4-methoxyphenyl)aldimines **104** in a one-pot process (Scheme 26) [101]. This pseudo-[3 + 2]-annulation reaction proceeds via a proline-catalyzed Mannich reaction/cyclization followed by an IBX-induced oxidative rearrangement to provide the final product **107**. A number of aldimines **104** decorated with electron-deficient substituents such as NO_2_, CN, CF_3_, F, Cl and Br worked well under optimized reaction conditions. Additionally, heteroaromatic aldehydes-based imines **104** furnished the desired pyrrole-2,4-dialdehydes **107** in good yields. Moreover, the practical utility of this method was examined through the gram-scale synthesis of **107**, the chemoselective functionalization of aldehyde groups at C2 and the synthesis of the medicinally important pyrrolo [3,2-*c*]quinoline scaffolds.

**Scheme 27:**
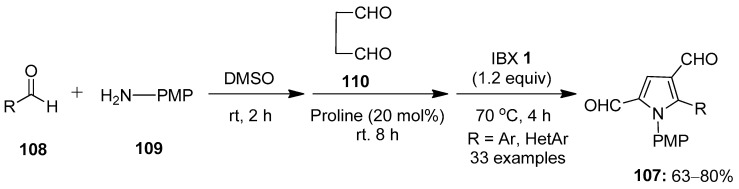
IBX-mediated synthesis of N-arylpyrrole-3-carbaldehydes **107** from in situ generated aldimines **104** and succinaldehyde **110** using proline as a catalyst.

In continuation, Kumar’s group demonstrated the one-pot multicomponent synthesis of N-arylpyrrole-3-carbaldehydes **107** via the in situ formation of aldimines **104** from aldehydes **108** and aromatic amines **109**, followed by sequential Mannich reaction–cyclization with succinaldehyde **110** and final IBX-mediated oxidative aromatization (Scheme 27) [102]. The scope of the reaction was examined with a variety of in situ generated aryl/hetero-aryl imines **104** to provide the corresponding products **107** in good yields.

**Scheme 28:**
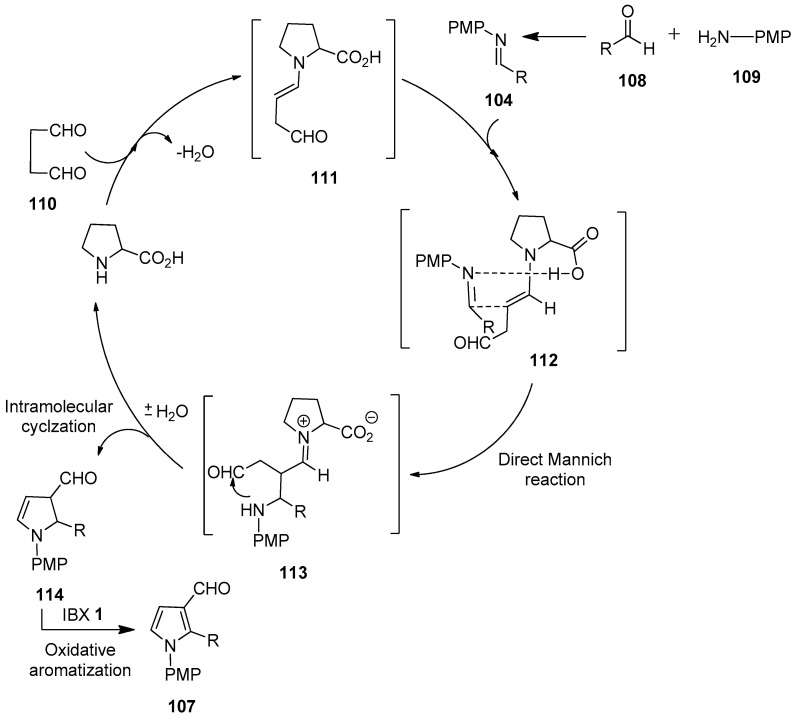
Proposed reaction mechanism for the IBX-mediated one-pot synthesis of N-arylpyrrole-3-carbaldehydes **107** using proline as a catalyst.

A stepwise mechanism proposed for the one-pot synthesis of N-arylpyrrole-3-carbaldehydes **107** is depicted in Scheme 28 [102]. Initially, the reaction of succinaldehyde **110** with the proline catalyst generates enamine **111**, which reacts with the in situ generated NPMP-imine **104** via a direct Mannich reaction to form the Mannich product **113**. Then, the intermediate **113** undergoes intramolecular cyclization to furnish dihydropyrrole **114**, along with the regeneration of the catalyst. Finally, the IBX-mediated oxidation of the cyclic enamine intermediate **114** affords pyrrole-3-carboxaldehyde **107**.

**Scheme 29:**
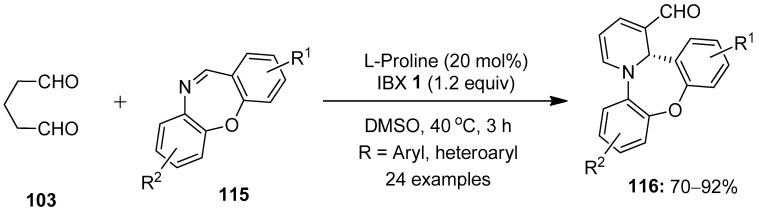
IBX-mediated synthesis of 1,4-oxazepines-fused 1,2-dihydropyridines **116** from glutaraldehyde **103** and cyclic imines **115**.

Dibenzo[*b*,*f*][1,4]oxazepine (DBO) derivatives are privileged scaffolds in organic chemistry, owing to their interesting medicinal and biological properties [103,104]. In this respect, Kumar’s group developed the synthesis of 1,4-oxazepines-fused 1,2-dihydropyridines (DHPs) **116** via a proline-catalyzed [4 + 2] annulation between glutaraldehyde **103** and cyclic imines **115** (Scheme 29) [105]. The reaction scope was explored with a variety of substituted dibenzoxazepine imines **115**, and the resulting products **116** were isolated in high yields (70–92%) with excellent enantioselectivity (up to >99:1 er). However, oxazepine-imines with o-CF_3_ substitution failed to provide the desired products, possibly due to steric hindrance caused by the CF_3_ group.

**Scheme 30:**
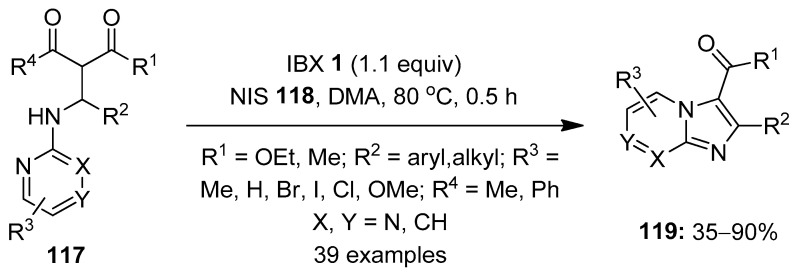
Oxidative cyclization of the Mannich precursors **117** to imidazo [1,2-*a*]-fused heterobicyclic scaffolds **119** using IBX **1** as an oxidant.

In 2019, Makra et al. developed the hypervalent iodine-mediated intramolecular oxidative annulation of Mannich precursors **117** towards the synthesis of imidazo [1,2-*a*]-fused heterobicyclic scaffolds **119** via a C–H functionalization/C–N bond formation strategy (Scheme 30) [106]. Among the tested oxidants, IBX **1** provided the highest product yield. A variety of Mannich precursors were treated with IBX **1** (1.1 equiv.) in the presence of NIS **118** (1.5 equiv.) as an additive in DMA to yield functionally diverse imidazo [1,2-*a*]-pyridine, -pyrimidine and -pyrazine scaffolds. Gratifyingly, the one-pot synthesis of the selected compounds **119** was achieved by reacting *β*-keto esters with primary aromatic amines and aldehydes in the presence of phosphotungstic acid (PTA) and IBX/NIS, providing overall yields up to 25%. The synthesized imidazo [1,2-*a*]pyridine motif (IPY) is a key structural unit in various bioactive compounds [107,108].

**Scheme 31:**
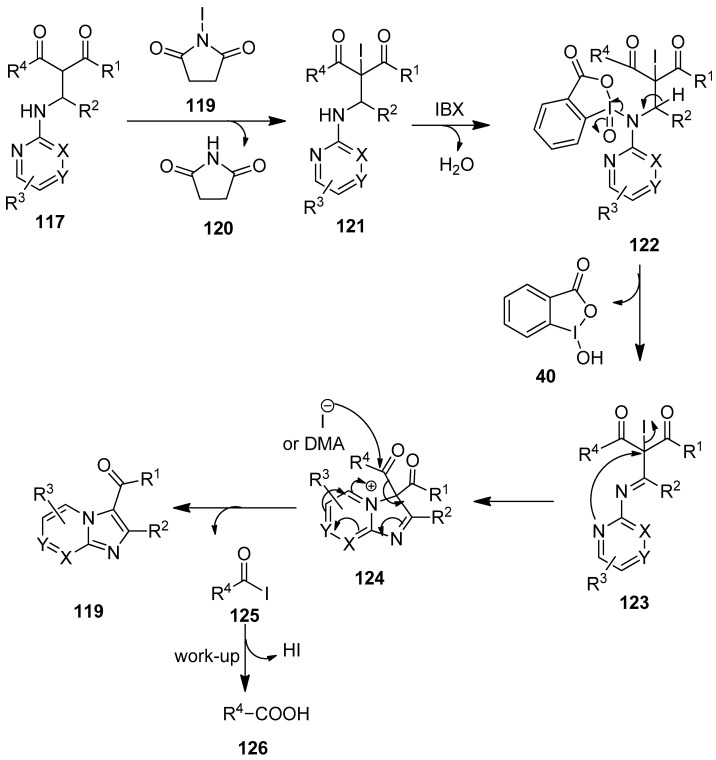
The plausible mechanism for the oxidative cyclization of the Mannich precursors **117** using IBX **1** as an oxidant.

A plausible mechanism for the oxidative annulation reaction of **117** is depicted in Scheme 31 [106]. The reaction begins with the α-halogenation of the Mannich precursor **117** with NIS **118** to afford the iodo intermediate **121**, followed by subsequent NH-oxidation with IBX **1**, yielding the intermediate **123**. Then the intermediate **123** cyclizes intramolecularly through the formation of a new C–N bond to produce the corresponding intermediate **124**. Finally, stabilization with the retro-Claisen–Schmidt reaction leads to the desired product **119**.

**Scheme 32:**
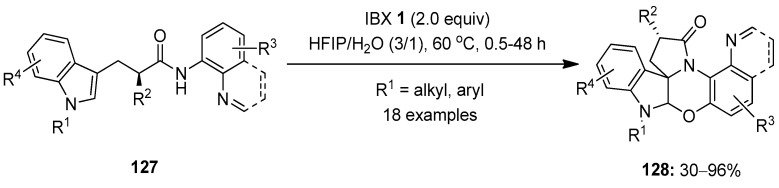
Tandem oxidation–cyclization of tryptophan analogs **127** to yield polycyclic spiroindolines **128** using IBX **1** as an oxidant.

In 2020, Zhang et al. demonstrated the IBX-mediated tandem oxidation–cyclization of tryptophan analogs **127** with *N*-arylamide side chains, producing a library of polycyclic spiroindolines **128** under mild conditions (Scheme 32) [109]. A number of *N*-protected tryptophan derivatives **127** worked well, and the anticipated oxazine-bearing complex polycyclicindolines **128** were synthesized in 30–96% yields. However, the *N*-unprotected tryptophan analog **127** (R^1^ = H) failed to yield the desired product. The key feature of this tandem cyclization reaction is the creation of multiple stereocenters, including a quaternary stereocenter, in a single step.

**Scheme 33:**
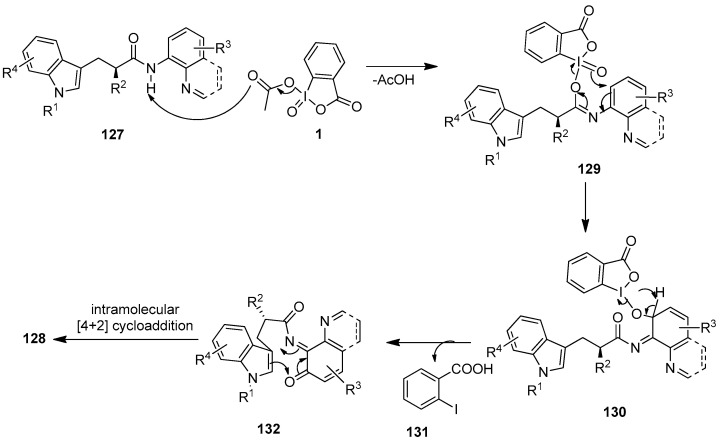
The proposed mechanism for the tandem oxidation–cyclization of tryptophan analogs **127** using IBX **1** as an oxidant.

The proposed mechanism for the IBX-mediated spiro-fused cyclization of tryptophan analogs **127** is shown in Scheme 33 [109]. The reaction initiates with the attack of an amide O atom on to the iodine center of IBX **1** to produce the iodoimidate intermediate **129** with the release of one AcOH molecule. Then, the nucleophilic oxo group on the iodine center of **129** intramolecularly attacks the ortho-position of the aminoquinoline (AQ) group, triggering the dearomatization of the aniline ring, followed by the cleavage of the O−I bond to yield the intermediate **130**. Deprotonation and subsequent cleavage of the O−I bond of **130** generates the o-iminoquinone intermediate **132** along with 2-iodobenzoic acid **131** as a by-product. Finally, o-imidoquinone **132** undergoes an intramolecular [4 + 2] cycloaddition to furnish polycyclic **128**. 

**Scheme 34:**
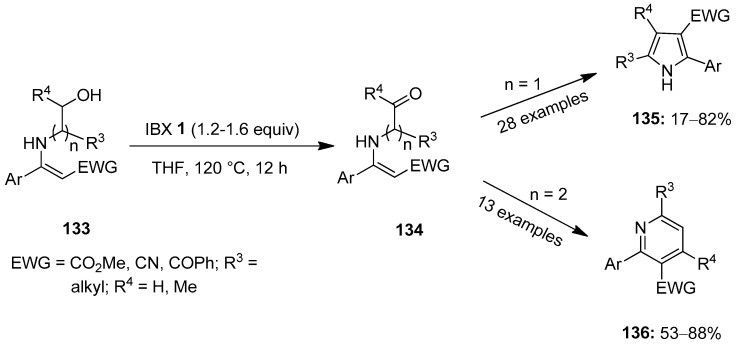
Oxidative cyclization of *N*-hydroxyethyl enamines **133** to yield 2,3-disubstituted pyrroles **135** and pyridines **136** using IBX **1** as an oxidant.

In the same year, Gao et al. presented an important method for the synthesis of 2,3-disubstituted pyrroles **135** through the IBX-mediated oxidative cyclization of *N*-hydroxyethyl enamines **133** (*n* = 1) via the intermediate **134** (Scheme 34) [110]. Phenyl enaminoesters substituted with methoxy, fluoro, chloro and bromo groups provided the desired pyrrole **135** in good yields. Likewise, substrates with electron-withdrawing groups such as CO_2_Me, -CO_2_Et, -CN and -COPh, were well tolerated under these conditions. Further exchanging the *N*-substituted moiety with *N*-hydroxypropyls (*n* = 2) yielded 2,3-disubstituted pyridines **136** in moderate to good yields.

**Scheme 35:**
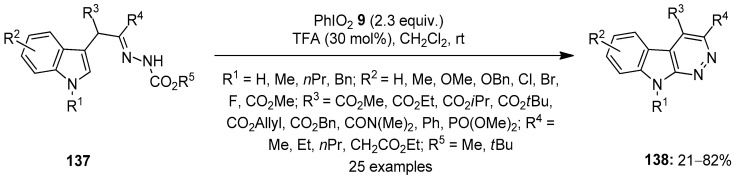
Oxidative cyclization of α-indolylhydrazones **137** to yield azacarbolines **138** using PhIO_2_ **9** as an oxidant.

In 2021, Favi and others developed an unprecedented method to access polysubstituted indolefused pyridazines **138** via the intramolecular oxidative cyclization of α-indolylhydrazones **137** using iodylbenzene (PhIO_2_) **9** as an oxidant (Scheme 35) [111]. The addition of TFA (20 mol%) was essential for the smooth proceeding of the reaction. The substrate scope of the cycloamination reaction was investigated with an array of α-indolylhydrazones **137,** and the anticipated azacarbolines **138** were obtained in good to excellent yields.

**Scheme 36:**
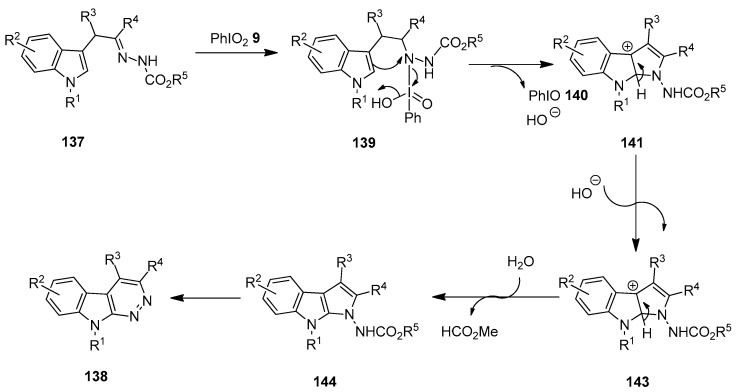
Tentative mechanism for the oxidative cyclization of α-indolylhydrazones **137** using PhIO_2_ **9** as an oxidant.

A proposed mechanistic pathway for the C(sp^2^)−H/N−H dehydrogenative coupling reaction of α-indolylhydrazones **137** is depicted in Scheme 36 [111]. The reaction begins with the oxidation of **137** by PhIO_2_ **9** to form the N-iodo intermediate **139** following CH/NH tautomerization. Subsequently, the intramolecular electrophilic cyclization of indole at C-2 with activated nitrogen generates the intermediate **141** with the release of PhIO **140** and HO−. The further deprotonation and aromatization lead to the key intermediate, pyrrolo [2,3-*b*]indole **143**. Finally, the hydrolysis of the intermediate **143** followed by ring expansion and oxidative aromatization affords the expected azacarboline product **138**.

## 9. Miscellaneous Reactions

**Scheme 37:**
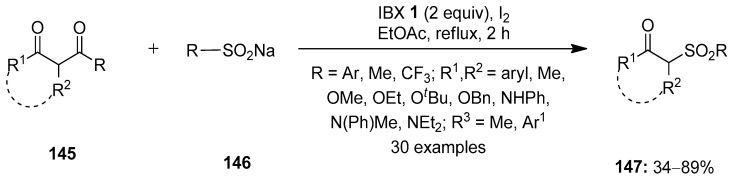
IBX–I_2_-mediated synthesis of β-carbonyl sulfones via the deacylative sulfonylation of 1,3-dicarbonyl compounds.

In 2016, Kuhakarn disclosed the deacylative sulfonylation of 1,3-dicarbonyl compounds **145** with sodium sulfinates **146** by employing IBX **1** and a catalytic amount of iodine (Scheme 37) [112]. This led to the one-pot synthesis of β-carbonyl sulfones **147** in good yields with a broad substrates scope. Notably, benzoylacetone derivatives **145** with electron-donating groups (Me, ^t^Bu, OMe) provided higher product yields compared to derivatives with electron-attracting groups (Cl and NO_2_). The reactions with acetylacetone, β-keto esters and β-keto amides as substrates yielded the corresponding products in low to moderate yields. The same group previously reported the synthesis of β-keto sulfones by reacting alkenes with sodium arenesulfinates in the presence of IBX–iodine [113].

## 10. Conclusions

This review summarized the recent developments in oxidative transformation reactions using hypervalent iodine(V) reagents. Hypervalent iodine compounds have emerged as versatile, non-toxic and environment friendly oxidants in organic synthesis. Although the chemistry of trivalent iodine reagents is well developed, the synthetic application of organoiodine(V) reagents has seen considerable growth only in recent times. Various synthetic transformations such as oxidation of alcohols, oxidation of amines, oxidation of amides, oxidation of aromatic compounds, oxidation of alkenes and oxidative cyclizations have been achieved using iodine(V) reagents. In particular, 2-iodoxybenzoic acid (IBX) and Dess–Martin periodinane (DMP) have received great attention owing to their mild oxidizing properties, high chemoselectivity and broad applicability. Moreover, significant work has been accomplished for the development of new catalytic systems based on in situ generated hypervalent iodine(V) reagents through the oxidation of organoiodine compounds. Addressing the solubility issues of IBX and designing new catalytic systems involving the in situ generation of hypervalent iodine(V) species represent an intriguing area of future investigation. In addition, the development of novel recyclable polymer-supported hypervalent iodine(V) reagents is a topic of great interest from a future perspective.

## Data Availability

Not applicable.

## Sample Availability

Samples of the compounds are not available from the authors.
